# Bacterial Cellulose-Alginate Composite Beads as *Yarrowia lipolytica* Cell Carriers for Lactone Production

**DOI:** 10.3390/molecules25040928

**Published:** 2020-02-19

**Authors:** Shuo Zhang, Huaying He, Shimin Guan, Baoguo Cai, Qianqian Li, Shaofeng Rong

**Affiliations:** Department of Biological Engineering, Shanghai Institute of Technology, No. 100 Haiquan Road, Fengxian District, Shanghai 201418, China; 13874474578@163.com (H.H.); guanshimin0000@163.com (S.G.); baoguocai@126.com (B.C.); lqq3486@163.com (Q.L.)

**Keywords:** gamma-decalactone, immobilisation, alginate, bacterial cellulose, *Yarrowia lipolytica*

## Abstract

The demand for natural lactone gamma-decalactone (GDL) has increased in the fields of food and cosmetic products. However, low productivity during bioprocessing limits its industrial production. In this study, a novel composite porous cell carrier, bacterial cellulose-alginate (BC-ALG), was used for long-term biotransformation and production of GDL. The effects of this carrier on biotransformation and related mechanisms were investigated. BC-ALG carriers showed improved mechanical strength over ALG carriers, with their internal embedded cell pattern changed to an interconnected porous structure. In five repeated-batch biotransformation experiments, the maximum concentration of GDL obtained in culture with BC-ALG carriers was 8.37 g/L, approximately 3.7 times higher than that from the medium with an ALG carrier alone. The result indicated that multiple hydrogen bonding interactions at the interface between BC and ALG contributed to the compatibility and stability of BC-ALG carriers. On the basis of the above results, the BC-ALG composite carrier can be considered ideal for immobilisation of cells for the production of GDL on an industrial scale, and has the potential to be utilised in other biological processes.

## 1. Introduction

Gamma-decalactone (GDL) is an important lactone additive with a peach-like flavour, and is widely used in foods, beverages and cosmetics [1]. Due to the drawbacks of limited yield and high cost of natural GDL traditionally obtained from plants and fruits, the production of GDL via biotransformation has attracted considerable research interest over the last decades. Biotransformation has the advantages of quality and safety identical to those of natural GDL, but at a lower cost [2,3]. For GDL biotransformation, *Yarrowia lipolytica* is reported to have the highest productivity among all strains [4]. Ricinoleic acid (RA), which is the main component of castor oil and its derivatives, is commonly utilised as a metabolic substrate [5,6,7,8]. RA is degraded through four β-oxidation cycles to form 4-hydroxy-decanoic acid, which is then circulated to GDL [9]. However, the yield of GDL decreases after a certain point, accompanied by a reduction in cell number during the bioprocess. This results from inhibitory effects of high concentrations of RA and the significant feedback effect of GDL, which lower both cell activity and GDL yield in long-term operations [10,11,12].

Many studies have shown that immobilised culture technology is effective in protecting cells and retaining their activity against inhibitors during the bioprocess [13,14,15]. In this case, immobilised cell systems with appropriate carriers could help cells tolerate unsuitable conditions better than free cell systems. Among different immobilisation techniques, the entrapment of cells within alginate (ALG) gel is a commonly used method because it is simple and maintains high cell viability and activity [16,17,18]. Lee et al. [19] compared different materials for immobilisation of *Sporidiobolus salmonicolor* to produce GDL and found that the most effective way was to immobilise cells in ALG, with a maximum concentration of 131.8 mg/L obtained after fermentation for 5 days. However, due to the poor mechanical properties and structural compactness of ALG beads, the carriers often led to leakage with limited production capacity. The chemical and physical properties of ALG could be improved by blending it with other materials. Zhao et al. [20] used a mixture of ALG and attapulgite to immobilise *Yarrowia lipolytica* and showed that productivity increased by 2.5 fold.

Bacterial cellulose (BC) is a kind of nanostructured cellulose synthesised by *Acetobacter xylinum*, which has many unique properties, including high porosity, high tensile strength, and low toxicity. It has been utilised as an ideal material to immobilise bacteria [21,22,23,24,25,26]. Żywicka1 et al. [27] immobilised *Saccharomyces cerevisiae* on BC and found that the amount of alcohol produced by immobilised cells was higher compared to that produced by free cells. Da-yu et al. [28] found that white rot fungi immobilised on BC exhibits improved efficiency and stability of pigment removal during wastewater treatment. In the present study, a BC-ALG-immobilized cell system was fabricated for GDL production. To obtain suitable immobilization carriers, characteristics of the carrier, such as pore structure, composition, mechanical strength, the optimal ratio of BC to ALG and the GDL productions by batch and repeated-batch biotransformation using BC-ALG culture were compared with those of free suspended culture (FSC) as well as immobilized culture (IC) entrapped within ALG were investigated.

## 2. Results

### 2.1. Optimum Composition of BC-ALG Carriers

To obtain a suitable BC-ALG carrier for GDL production, BC-ALG carriers with different compositions (ALG%: (ALG: BC): CaCl_2_%) were utilised for repeated batch biotransformation experiments.

**Figure 1:**
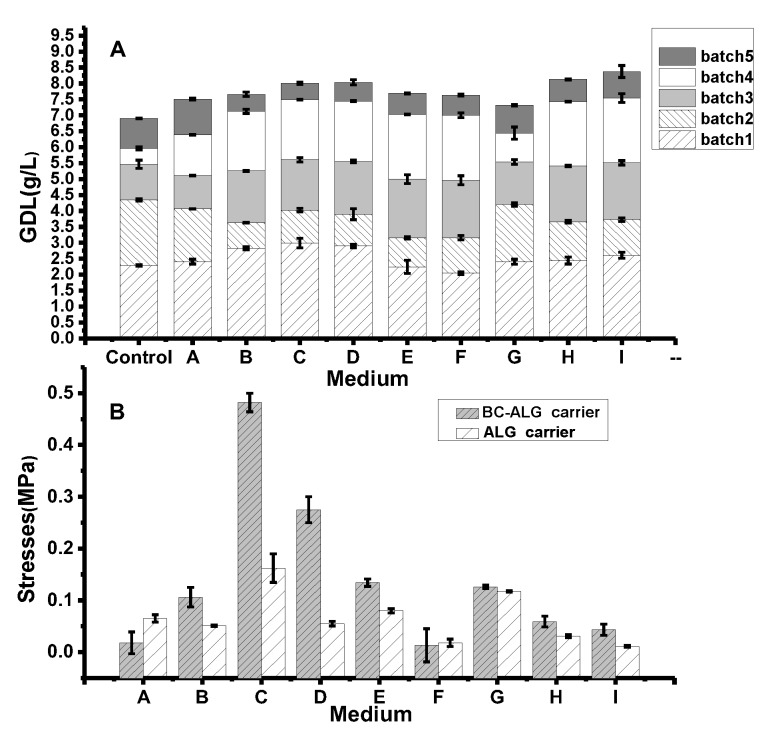
Comparison of gamma-decalactone (GDL) production based on different BC-ALG carriers (**A**) and the compressive strength of BC-ALG and ALG carriers (**B**). The group names A–I represent the carrier with different compositions according to Table 1.

The results are shown in Figure 1A. The GDL yield obtained in the broth was compared with those of different BC-ALG carriers with FSCs as controls. The GDL yield from five repeated-batch biotransformation experiments with BC-ALG carriers ranged from 7.33 to 8.37 g/L, which was higher than that in FSCs (6.91 g/L). In the first batch of biotransformation, the maximum GDL obtained in medium C was 2.99 g/L, which was 30% higher than that from FSCs. From the second to the fourth batch of biotransformation, the GDL yield from FSCs decreased gradually from 2.06 to 0.49 g/L while the GDL yield in the medium with BC-ALG carriers still reached 2.04 g/L. The maximum GDL yield was obtained in medium I. According to the results analysed by SPSS, the concentration of ALG (*p* = 0.032, < 0.05) and the proportion of ALG/BC (*p* = 0.029, < 0.05) were significantly correlated with the production of GDL.

To investigate the role of BC on the physical characteristics of the carriers, the mechanical strength was compared between BC-ALG and ALG carriers. As shown in Figure 1B, the incorporation of BC enhanced the mechanical strength of the carrier. At 2% ALG concentration (test numbers A, B and C), the compressive strength of carriers improved as the proportion of BC increased. However, when the concentration of ALG increased by 3% and 4%, the strength decreased. The stress per unit area of the ALG carrier ranged from 0.01 to 0.16 MPa, whereas that of the BC-ALG carrier was from 0.02 to 0.48 MPa. The mechanical strength of BC-ALG carriers was higher than that of ALG carriers.

For ALG-iemmobilized cells, the mechanical strength of those tested at 4.5% CaCl_2_ concentration (test numbers C, E and G) was higher than that of other test groups that were made by same ALG concentration. It is indicated that improving the CaCl_2_ concentration is a method to rapidly increase the mechanical strength of the pellet.

According to the reference standard of the yield of GDL, the optimum condition for preparing carriers was obtained in medium I: 4% ALG mixed with BC (according to the ratio of 2:1) crosslinked in 3% CaCl_2_. The BC-ALG carriers prepared through this method could be utilised at least five times, with GDL production 21% higher than that in FSC culture.

### 2.2. Repeated-Batch Biotransformation

**Figure 2:**
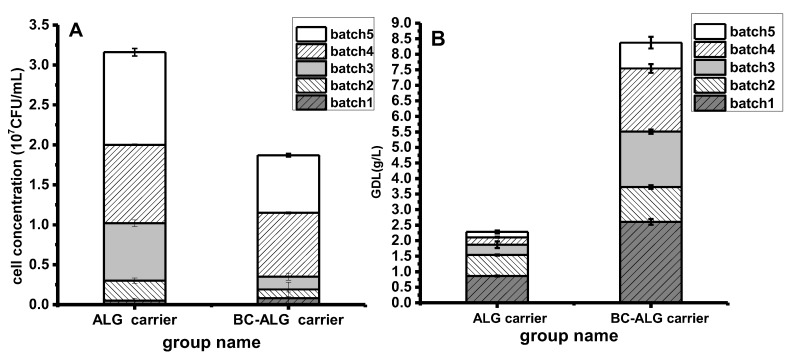
Comparison between medium with ALG and BC-ALG carriers in terms of cell leakage (**A**) and GDL yield (**B**) over 5 repeated-batch biotransformation experiments.

In this study, the results obtained from medium with BC-ALG carriers were compared with those from the ALG carrier. The amount of cell leakage was determined to evaluate the immobilisation ability of carriers. As shown in Figure 2A, In the first batch of fermentation, the BC-ALG system had slightly more cell leakage than ALG system, 0.08 × 10^7^ CFU/mL and 0.05 × 10^7^ CFU/mL, respectively. However, in the subsequent batches, the amount of cell leakage in the BC-ALG system was much lower than that in the ALG system. Finally, the amount of leakage by cells in the medium with BC-ALG carriers was 1.87 × 10^7^ CFU/mL, which was 40% less than that from the medium with ALG carriers. This phenomenon indicated that the BC-ALG carrier had a better structure to embed cells internally.

In addition, the total GDL concentration in the system was detected after each batch converted 48 h later, with average productivity (g/(L h)) to assess the merits of the two kinds of carrier in biological transformation process of GDL. As shown in Figure 2B, the average productivity of GDL was 0.0095 (g/(L h)) in the medium with ALG carriers, which was only 27.2% of that from medium with BC-ALG carriers during the five repeated-batch biotransformation experiments. The accumulated yield of GDL in the medium with BC-ALG carriers reached 8.37 g/L, which was 3.7 times higher than that from the medium with ALG carriers.

### 2.3. Characteristics of ALG and BC-ALG Carriers

**Figure 3:**
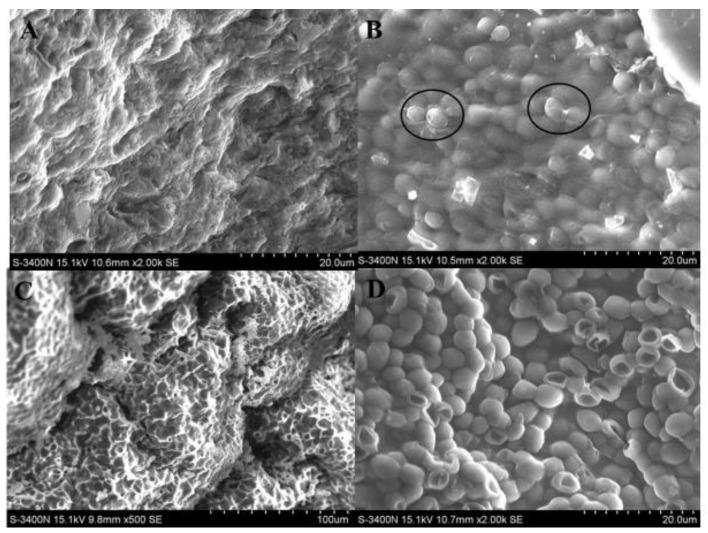
SEM images of ALG carriers (**A**), ALG carriers with cells (**B**), BC-ALG carriers (**C**) and BC-ALG carriers with cells (**D**).

SEM images of the ALG and BC-ALG carriers alone and those with *Y. lipolytica* embedded inside are shown in Figure 3. As illustrated in Figure 3A, the surfaces of the ALG carriers showed calcium alginate fibres being stacked together. Cells were completely and tightly encapsulated inside the carriers (Figure 3B, the boundary between the cell and the carrier can be clearly seen in the circle). In contrast, BC-ALG carriers had obvious concave and convex structures on the surface with interconnected hollow spaces in the interior, which is related to the holes formed by the unique 3D network of BC (Figure 3C). The cells were agglomerated in hollow spaces and were more exposed to the medium (Figure 3D). This finding revealed that the addition of porous BC in the carrier changed the original ‘stacked’ encapsulated cell pattern of the ALG carriers into ‘filled’ embedded cell patterns.

A comparison of our results showed that BC-ALG carriers were more stable as they retain overall carrier size and a relatively higher GDL productivity of 0.035 (g/(L h)) for the entire biotransformation, with less cell leakage.

**Figure 4:**
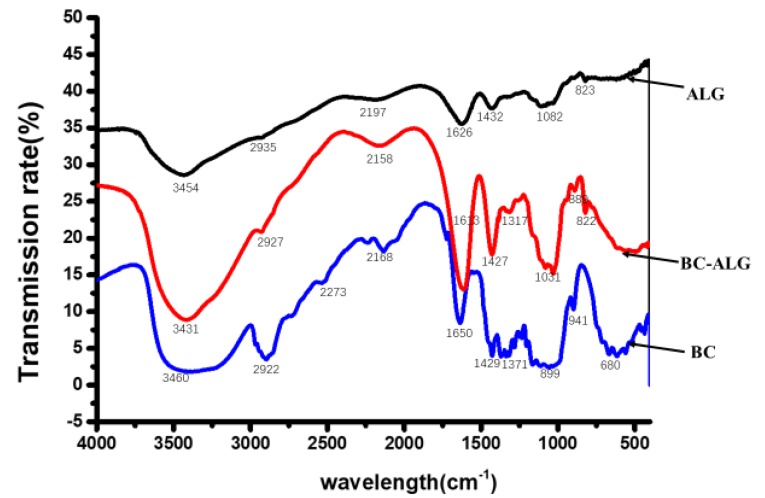
FTIR spectra of ALG, BC-ALG and BC.

FTIR was used to investigate potential interactions between ALG and BC in BC-ALG carriers (Figure 4). The FTIR spectra of ALG, BC-ALG and BC were measured at wavelengths of 4000–400 cm^−1^. Pure ALG showed characteristic peaks of asymmetric and symmetric carboxyl stretching bands centred at 1626 and 1432 cm^−1^, respectively. Additionally, a characteristic vibration peak of a sugar ring centred at 1082 cm^−1^ was observed. The strong and broad absorption peak at 3454 cm^−1^ was that of V(O-H). In contrast, the characteristic band of glucose carbonyl of BC was observed at 1650 cm^−1^, which was shifted to 1618 cm^−1^ for BC-ALG. These results indicated that certain changes had taken place in the spectra of ALG and BC after they were mixed and crosslinked with CaCl_2_. The corresponding absorption peak for BC-ALG moved in the lower wavenumber direction, indicating that the interaction between the two substances may be due to increased numbers of intermolecular hydrogen bonds in the structure of BC-ALG. However, no new absorption peaks appeared in the vibration peak of the BC-ALG composite fibre, which indicated that no other chemical reaction had taken place.

### 2.4. Comparison between FSC and BC-ALG Carriers in One-Batch Biotransformation

**Figure 5:**
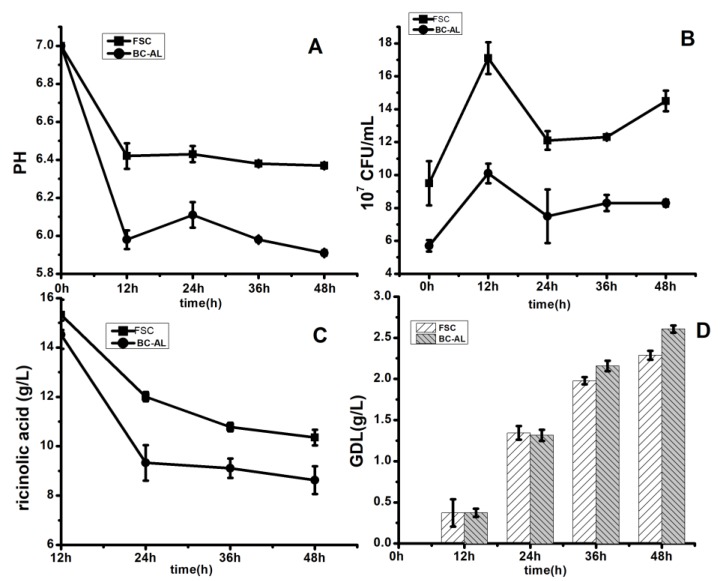
Changes in pH (**A**), cell growth (**B**), ricinoleic acid RA (**C**), and GDL concentration (**D**) in one-batch biotransformation with free suspended culture (FSC) and BC-ALG carriers.

Changes in pH values, cell proliferation profiles and RA and GDL concentrations between BC-ALG carriers and FSCs in one-batch biotransformation were compared. In terms of pH, both media showed no obvious differences. The pH decreased from 7.0 to 6.3 and 6.0, between 0–12 h, in the medium with FSCs and BC-ALG carriers, respectively (Figure 5A). Subsequently, the pH remained stable but with a lower value in the medium with the BC-ALG carrier.

In the 30 mL medium containing the 3 mL BC-ALG cell mixture, the total number of cells T was 9.8 × 10^7^ CFU/mL. The number of cells in the calcium chloride solution U was 3.9 × 10^7^ CFU/mL. The number of cells in BC-ALG pellets P was 5.85 × 10^7^ CFU/mL. According to the formula, the embedding rate of BC-ALG was 59.7%, the unembedding rate was 39.8%, and the sum of the two was 99.5%. Exclude the mixture remaining on the walls of syringes and other containers, the results suggested that the sodium citrate solution in this experiment had little effect on the viability of cells. According to the cell count measured in the BC-ALG-immobilised pellet solution, the number of cells inside the BC-ALG carrier was 5.7 × 10^7^ CFU/mL, and that for FSCs was 9.5 × 10^7^ CFU/mL because the embedding percentage was 60%, approximately. In the first 12 h, the proliferation rate increased by 80%, that is, 10.1 × 10^7^ CFU/mL and 17.1 × 10^7^ CFU/mL in the BC-ALG carriers and FSCs media, respectively, as shown in Figure 5B. However, after 12 h, a slight decline was observed, which lasted until 48 h. The final concentration of cells in the BC-ALG carrier and FSC medium was 8.3 × 10^7^ CFU/mL and 14.5 × 10^7^ CFU/mL, respectively, which showed an approximately 50% enhancement compared with the original media. The above results indicated that BC-ALG carriers had no obvious negative effects in terms of cell division compared with free cells.

As shown in Figure 5C, the highest consumption of RA took place between 0–12 h. RA decreased to 14.5 and 15.3 g/L from 25 g/L in the medium with BC-ALG carriers and FSCs, respectively. The yields of GDL in FSC and BC-ALG medium were 2.29 and 2.61 g/L, respectively, with a similar conversion rate of 16% (Figure 5D). However, the productivity of the cell units was not the same. The average productivity of GDL per 10^7^ unit cells in the FSC and immobilised pellet system was monitored every 12 h. During biotransformation, the average productivity of GDL in the FSC system was 0.0437 ± 0.03 mg, whereas that in the BC-ALG immobilisation system was 0.0813 ± 0.02 mg, which was approximately two-fold higher than in FSCs (Sig = 0.034, *p* < 0.05). This result indicated that BC-ALG carriers could protect cells and increase the productivity of GDL.

The results also show that BC-ALG carriers had no negative effects on the embedded cells, and the tolerance of such cells to otherwise unsuitable conditions could be improved, resulting in higher productivity of the cell unit. In addition, this effect is supposed to be more significant in repeated-batch biotransformation.

### 2.5. Cell Survival Rate

In the medium without GDL, the numbers of viable cells in the FSC- and BC-ALG-immobilised systems were 11.6 × 10^6^ CFU/mL and 6 × 10^6^ CFU/mL, respectively. In comparison, in the medium with GDL, the numbers were 1.4 × 10^6^ CFU/mL and 4 × 10^6^ CFU/mL in the FSC- and BC-ALG-immobilised systems, respectively. It was revealed that after treatment with GDL, the survival rate was only 12.065 ± 0.024% in the FSC system, which was lower than 66.7 ± 0.005% in the BC-ALG-immobilised system. This indicated that the cell survival rate was greatly improved in the BC-ALG-immobilised system, and this advantage was also observed in the production of GDL in repeated fermentation experiments described above.

## 3. Discussion

In this study, a novel biocompatible porous cell carrier was formulated with BC and ALG to improve the production of GDL. The BC-ALG-immobilized cells showed obvious advantages over free cells in batch transformation after being repeated five times. The results of repeated-batch fermentation showed that BC-ALG-immobilised cells can maintain 240 h of production activity, and that the BC-ALG carrier could be utilised over at least five repeated cycles without any change to its structure. The maximum GDL concentration obtained in culture with BC-ALG carriers after five repeated-batch biotransformation experiments was 8.37 g/L. This concentration is also much higher than that obtained from media with other immobilisation methods, as reported previously. Adelaide et al. [29] compared GDL production by *Y. lipolytica* immobilised in different materials and obtained a maximum yield of 1.597 ± 34 mg/L from cells adsorbed on polymethacrylate after 264 h. The GDL yield of 482 mg/L, which was obtained from *Y. lipolytica* immobilised in a mixture of polyvinyl alcohol and carrageenan after a 24 h biotransformation, was much lower than that obtained in the medium with the BC-ALG carrier [30].

In the BC-ALG carrier, BC is supposed to improve the poor characteristics of dense nonuniform structures and rapid degradation of ALG carriers during biotransformation [31,32,33,34,35]. Żywicka et al. found that the porosity-related parameters, including specific surface area, pore size, pore volume, and different mechanical strength, have a significant impact on the cell immobilization [36,37,38]. The SEM images showed that the polyporous structure of the BC-ALG carriers provides much more space for the cells to grow and have greater opportunity to be exposed to the external medium, which facilitates higher mass transfer rates and results in relatively higher GDL yields. On the other hand, BC can also improve the mechanical strength of the carrier, making BC-ALG more durable than the ALG carrier. This mechanism may be caused by the interconnected porous structure of the BC-ALG carrier, which resulted in strong interactions between BC and ALG, was suitable for immobilising cells and facilitating mass transfer. The structure of cellulose, consisting of small pores (240–260 nm) distributed on the surface, and large pores (400–450 nm) distributed in the interior, was reported to be effective for cell immobilisation [39].

Aguado et al. [40] found that there was growth inhibition on the cells when the concentrations of GDL was much more than 200 mg/L. In addition, when the concentration of GDL reached 500 mg/L, there was a cytotoxic effect on the cells. In this study the GDL concentration produced by a 48 h transformation was more than 2 g/L, which may have toxic effects on cells and lead to cell death. As expected, the results from cell survival rate experiments proved that cells immobilized in BC-ALG carriers were more tolerant of high concentrations of GDL than free cells. The survival rate of free cells in the FSC system was 12.065% ± 0.024, while it was 66.7% ± 0.008 in the BC-ALG-immobilized system, which indicated that the survival rate of cells in BC-ALG-immobilized system was greatly improved. Furthermore, the productivity of cell units in BC-ALG carriers for the production of GDL was two-fold higher than that of free cells. This relatively high productivity remained stable during the entire biotransformation process, with a cell leakage of 1.87 × 10^7^ CFU/mL, which was 18.51% of the total number of cells immobilized in BC-ALG carriers. This indicates that cells were much more resistant to high concentrations of RA and to the feedback inhibitory effects of GDL compared with free cells.

## 4. Materials and Methods

### 4.1. Microorganism and Materials

*Yarrowia lipolytica* (CGMCC 2.2087) used for GDL biotransformation was obtained from the China General Microbiological Culture Collection Center, Beijing, China. BC was purchased from Guilin Qihong Technology Co., Ltd. (Guilin, Guangxi Province, Chana). RA substrate was obtained from Zibo Zhoucun Mingdong Chem, Ltd. (Zibo, Shandong, China), and GDL standard was provided by Adamas (Shanghai, China). Analytical grade n-butyl alcohol and other chemicals were purchased from Sinopharm Chemical Reagent Co., Ltd. (Beijing, China).

### 4.2. Cultivation of Microorganisms

Preinoculum of *Y. lipolytica* (CGMCC 2.2087) was prepared by transferring a colony grown on agar culture medium into 50 mL of sterilised liquid culture medium containing 10 g/L glucose, 10 g/L peptone and 12 g/L yeast extract. The pH of the media was adjusted to 6.0 by adding 2 mol/L sodium hydroxide and 2 mol/L hydrochloric acid. After 24 h of culture with 200 rmp agitation at 28 °C, *Y. lipolytica* cells in the exponential growth phase (the number of cells reached 10^8^ CFU/mL) were prepared to be immobilised or inoculated into the biotransformation medium.

### 4.3. Preparation of Cell Carriers

**Figure 6:**
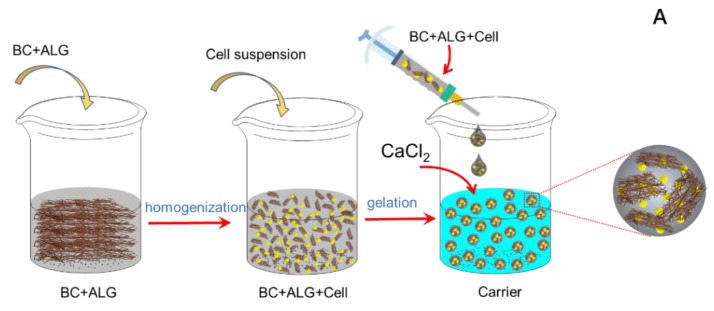

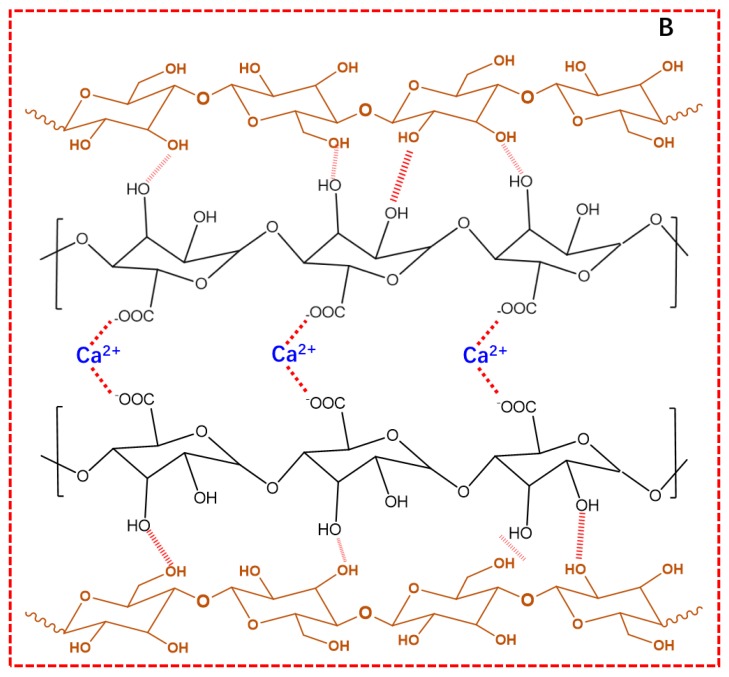

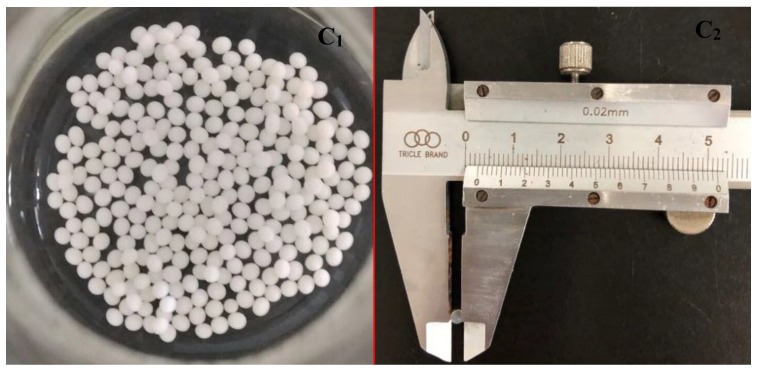
Procedure for the preparation of bacterial cellulose-alginate (BC-ALG) carriers (**A**), the mechanism of the interaction of the components inside (**B**) and pictures of the carriers with immobilized cells (**C_1_**,**C_2_**).

(1) The BC-ALG carriers were prepared via a three-step method: (1) the purified BC fibres were homogenised in a blender and then mixed with ALG solution at different ratios (ALG: BC = 4:1; ALG: BC = 3:1; ALG: BC = 2:1). (2) A total of 10 mL of Y. lipolytica cells obtained in Section 2.2 was centrifugated at 4 °C at a speed of 5000 rpm for 5 min. The cells were resuspended in 1 mL 0.9% NaCl solution to form cells suspension with concentration of 10^9^ CFU/mL. Then, 5 mL of cells suspension as above was centrifugated and resuspended in 1 mL 0.9% NaCl solution to form the final cell solution. (3) A total of 40 mL of mixture of fibres and ALG from step (1) and 10 mL final cell solution from step (2) were mixed according to the proportion of 4:1, and then it was filled into the 10 mL sterile syringe to drop into a solution of calcium chloride to form the immobilized cells for 2 h with natural pH in room temperature. The shape of the cell carrier was a bead with the size of 2.50 ± 0.2 mm (Figure 6C_1_,C_2_). The carriers were rinsed with sterile physiological saline twice to remove excess Ca^2+^. The preparation process is shown in Figure 6A.

BC-ALG-immobilized pellets were prepared according to Table 1. And ALG immobilized pellets were prepared according to Table 2.

The preparation principle is primarily due to the fact that Ca^2+^ and other bivalent cations tend to bind to the G-region of the ALG molecule, which is rich in gulaururonic acid. The carboxyl groups on the linked polymer chains are connected by hydrogen bonds, while the polymer chains are bound by the formation of multivalent bonds with Ca^2+^, which is shown in Figure 6B.

To investigate the effects of ALG, BC, and CaCl_2_ on the biotransformation performance of carriers, an orthogonal experiment was designed and the data were analysed by Statistical Product and Service Solutions (SPSS) software. The program of orthogonal experiments is listed in Table 1. It was divided into nine groups A-I. A, B and C, representing the concentration of ALG, the proportion of ALG/BC (*v*/*v*) and the concentration of calcium chloride.

### 4.4. Comparison of Productivity between Cells Immobilised in BC-ALG and ALG Carriers

Immobilised cells prepared with optimal proportions of ALG, BC, and CaCl_2_ were added to 30 mL of biotransformation medium containing 7 g/L K_2_HPO_4_, 7 g/L MgSO_4_·7H_2_O, 0.1 g/L l-carnitine, 10 g/L glycerine, 1 g/L CaCl_2,_ and 25 g/L RA in a 250 mL Erlenmeyer flask. Further, the solution was incubated for 48 h at 30 °C with agitation of 250 rpm. For repeated-batch biotransformation, the broth was withdrawn after 48 h of each batch. The amount of extracted GDL and number of cells that leaked were determined. Subsequently, the cell carriers were transferred into fresh medium to start the next batch. This procedure was repeated until GDL yields dropped sharply. These results were compared with those of the free suspended culture (FSC) and ALG carriers, which were taken as references. For FSCs, the number of cells was determined after each batch, and the cells were collected by centrifugation for the next batch. During biotransformation, changes in cell leakage and GDL concentrations were monitored every 48 h for each batch. All experiments were performed in triplicate.

### 4.5. Analytical Methods

#### 4.5.1. Cell Concentration Measurement

Total yeast count was performed by serial dilutions followed by spread plating over the surface of sterile potato dextrose agar plates with slight modifications [41]. Cell counting with immobilized carriers: (1) Immobilized cells were washed twice with sterile physiological saline before being dissolved in 30 mL (the same fermentation broth volume) 5% sterile sodium citrate in a 250 rpm shaker to accelerate the dissolution of BC-ALG pellets at 30 °C. (2) The resulting suspension was serially diluted from 10-1 to 10-6. And then 0.1 mL of each dilution was used for plating count [29]. The plates were incubated at 30 °C for 48 h. The results were expressed as colony-forming units per mL (CFU/mL) based on the average count of triplicate samples.

In order to detect the effect of sodium citrate on the cell viability, the amount of the cells in different situations was calculated. (1) Before the immobilization, the amount of cells in the mixture of BC-ALG and cells was determined as T. (2) Then, it was dropped into the 30 mL calcium chloride solution and immobilized for 2 h and the number of cells suspended in the calcium chloride solution was determined as U. (3) The immobilized pellets were dissolved in 30 mL sodium citrate solution and the cell count P was measured. The embedding rate of the BC-ALG carrier is calculated as follows:

Embedding rate = P/T

The unembedded rate of calcium chloride solution is calculated as follows:

Unembedding rate = U/T

Finally, the difference between the sum of embedding rate and unembedding rate with 1 is compared.

#### 4.5.2. pH Measurement

A pH meter (PHS-3C, Leici, Shanghai, China) was used to determine the pH of samples collected from the culture every 12 h in triplicate.

#### 4.5.3. Analysis of Components in Medium

Pure chemical compounds (≥98%) were used as standards. The RA and GDL concentrations in the broth and those extracted from BC-ALG and ALG carriers were measured. The structure of the carrier was destroyed by squashing before extraction, in which n-butanol was added as the extractant. Subsequently, the sample was centrifuged at 1000 rpm for 10 min, and then the supernatant was filtered and injected into an analysis bottle for testing.

A gas chromatograph (Agilent 6890 N, Agilent Technologies, Ltd., Santa Clara, CA, USA) equipped with a 19091 J-433 HP-5 chromatographic column was utilised to analyse GDL concentrations. The split ratio was 80:1, and N_2_ was the carrier gas. The operating conditions for the analysis were as follows: 2 μL of sample was injected at 250 °C and the detector temperature was set to 300 °C. The oven temperature was maintained at 120 °C for 2 min and then increased to 205 °C at a rate of 30 °C/min. It was further increased to 215 °C at a rate of 4 °C/min and finally set to 280 °C at a rate of 20 °C/min, which was held constant for 3 min.

An HPLC instrument (Agilent 1260, Agilent Technologies, Ltd., Santa Clara, CA, USA) equipped with an XDB-C18 column (250 × 4.5 mm) was utilised to determine the concentration of RA. Approximately 2 μL of the sample was injected into the column and eluted at a flow rate of 1 mL/min. The mobile phase was composed of 5% *v*/*v* 0.1% phosphoric acid and 95% *v*/*v* acetonitrile. The detection wavelength was 205 nm.

The conversion rate of the product was estimated as follows:

$$\mathrm{Conversion}\;\mathrm{rate}\left(\%\right)=\frac{\frac{\mathrm{The}\;\mathrm{concentration}\;\mathrm{of}\;\mathrm{GDL}}{\mathrm{MW}\;\mathrm{of}\;\mathrm{GDL}}}{\frac{\mathrm{The}\;\mathrm{concentration}\;\mathrm{of}\;\mathrm{the}\;\mathrm{converted}\;\mathrm{RA}}{\mathrm{MW}\;\mathrm{of}\;\mathrm{RA}}}\times 100$$

where MW was the molecular weight.

The average productivity was estimated as follows:

$$\mathrm{average}\;\mathrm{productivity}=\frac{C_{\mathrm{GDL}}}{T}$$

where C was the concentration, T was fermentation time (48 h).

#### 4.5.4. Cell survival Measurement

*Y. lipolytica* cells as FSCs and immobilised in BC-ALG carriers were inoculated into bioconversion medium containing 2 g/L of GDL at 30 °C for 48 h; simultaneously, the same amount of *Y. lipolytica* cells and BC-ALG-immobilised cells were inoculated into a bioconversion medium without GDL as a control. After 48 h of cell culture, the numbers of surviving cells in both the media were measured: (1) the immobilised cells were washed twice with sterile physiological saline and then dissolved in 5% sterile sodium citrate to generate a cell suspension, (2) FSCs were taken directly from the medium as a cell suspension, and (3) 0.1 mL of each cell suspension was used for culture plate colony counting. The plates were incubated at 30 °C for 48 h. The results are expressed as Colony-Forming Units (CFU/mL) in triplicate. The cell survival rate was calculated according to the following formula:

$$\mathrm{Cell}\;\mathrm{survival}\;\mathrm{rate}\;=\frac{\mathrm{Total}\;\mathrm{number}\;\mathrm{of}\;\mathrm{viable}\;\mathrm{cells}\;\mathrm{in}\;\mathrm{GDL}\;\mathrm{medium}}{\mathrm{Total}\;\mathrm{number}\;\mathrm{of}\;\mathrm{cells}\;\mathrm{without}\;\mathrm{GDL}\;\mathrm{medium}}\times 100\%$$

### 4.6. Characterization

#### 4.6.1. SEM

The structural properties of ALG- and BC-ALG-immobilized cells and their carriers before fermentation were observed by SEM (Hitachi S-3400N, Tokyo, Japan). After being washed and freeze-dried, the sample was fixed to the stage with a carbon film conductive paste, and conductive gold powder was deposited by ion sputtering under a vacuum. The microstructure of the carriers and images of *Y. lipolytica* were observed by SEM at an accelerated voltage of 15.0 KV.

#### 4.6.2. Measurement of Mechanical Properties

BC-ALG carriers were prepared according to the conditions mentioned in the section “*Preparation of cell carriers*” (Section 2.3). ALG carriers were prepared under the same conditions but without BC. The prepared BC-ALG and ALG carriers were subjected to compressive strength testing.

A SUN500 universal material machine (Cardano al Campo, Co., Varese, Italy) was utilised to determine the compressive strength of the carriers. The diameter of the carriers was measured with a digital Vernier calliper. A force value of 50 N in the sensor was selected on the cantilever. The conditions of the compressive tests were as follows—the clamping distance was 2.8 mm and the crosshead speed of the load cell was 1 mm/min. The temperature was set to 25 ± 2 °C with a relative humidity of approximately 50% ± 5%. The test was repeated five times for each sample.

#### 4.6.3. FTIR

FTIR spectroscopy is commonly used to determine the structure of compound regarding the special functional groups and chemical bond. In this study, an FTIR spectrometer (Shimadzu, Kyoto, Japan) was used to analysis the structure of ALG, BC and BC-ALG in the 4000–400 cm^−1^ range.

## 5. Conclusions

In this study, a novel composite porous carrier, BC-ALG, was used for long-term biotransformation of GDL. The carriers can be utilised for at least five repeated batches, with the highest production of 8.37 g/L and with fewer cell leakages. Compared with the production process of the FSC system and the commonly utilised ALG-immobilised system, the combination of porous BC improved the structure of carriers for embedding cells in the long-time production of GDL as an industrial process, and it also has the potential to be utilized in other bioprocesses.

## Figures and Tables

**Table 1:** The different composition of the BC-ALG carriers.

Test Numbers	(A) ALG%	(B) ALG:BC	(C) CaCl_2_%
A	2	4	1.5
B	2	3	3.0
C	2	2	4.5
D	3	4	3.0
E	3	3	4.5
F	3	2	1.5
G	4	4	4.5
H	4	3	1.5
I	4	2	3.0

**Table 2:** The different composition of ALG carriers

Test Numbers	ALG%	CaCl_2_%
A	2	1.5
B	2	3.0
C	2	4.5
D	3	3.0
E	3	4.5
F	3	1.5
G	4	4.5
H	4	1.5
I	4	3.0

## Abbreviations

gamma-decalactone (GDL), ricinoleic acid (RA), bacterial cellulose (BC), alginate (ALG), bacterial cellulose–alginate (BC–ALG), free suspended culture (FSC), *Yarrowia lipolytica* (*Y. lipolytica*), Scanning electron microscopy (SEM), high-performance liquid chromatography (HPLC), Fourier transform infrared spectroscopy (FTIR).
